# Modeling intraindividual means and variances from ecological momentary assessment data: comparing standard computational formulas to mixed-effects location-scale model estimates

**DOI:** 10.1007/s10865-025-00628-0

**Published:** 2026-05-08

**Authors:** Wei-Lin Wang, Chih-Hsiang Yang, Rachel Nordgren, Jixin Li, Stephen Intille, Genevieve F. Dunton, Donald Hedeker

**Affiliations:** 1 Department of Population and Public Health Sciences, University of Southern California, 1845 N. Soto St., Los Angeles, CA 90032, USA; 2 Department of Exercise Science, Arnold School of Public Health, University of South Carolina, Columbia, USA; 3 Department of Public Health Sciences, University of Chicago, Chicago, USA; 4 Khoury College of Computer Sciences and Bouvé College of Health Sciences, Northeastern University, Boston, USA; 5 Department of Psychology, University of Southern California, Los Angeles, USA

**Keywords:** Mixed-effects location scale model, Intraindividual variability, Ecological momentary assessment, Longitudinal data analysis methods, Missing data

## Abstract

Traditionally, intraindividual means and variances derived from ecological momentary assessment (EMA) data have been calculated using standard computational formulas (SCF), such as subject-level means and standard deviations. However, these SCF methods assume uniform precision across subjects, disregarding variation in the number of observations, missing data issues, and the non-continuous nature of data scales. This study evaluated the predictive accuracy of the coefficients of intraindividual means and variances computed via SCF against those estimated using random effects from a Mixed-Effects Location Scale (MELS) model. A five-scenario simulation study was conducted: (1) varying numbers of observations per subject, (2) varying mean-to-variance ratios, (3) varying proportions of missing data under a missing completely at random (MCAR) assumption, (4) varying proportions of missing data under a missing at random (MAR) assumption, and (5) varying categories of ordinal scale responses. Bias and coverage of the mean levels and variability coefficients were compared across methods. In addition, a real-life dataset was used to compare the difference of means and variances between SCF and MELS approaches. Results consistently showed that the MELS model approach outperformed SCF method, yielding lower bias and higher coverage of the coefficients across all scenarios. These findings support the use of MELS for more accurate and reliable estimation of intraindividual means and variances in EMA data, highlighting its advantages for subsequent predictive modeling.

## Introduction

Ecological Momentary Assessment (EMA) data, which are repeatedly collected in real-time and real-world settings via mobile devices, have increasingly gained prominence in behavioral sciences for measuring individuals’ situational, psychological, behavioral, and physiological states (Piot et al., 2022; Trull & Ebner-Priemer, 2009). Unlike cross-sectional or single time-point assessments, dense EMA data provide a robust framework for capturing nuanced and fluctuating experiences of individuals within their natural environments (Burke et al., 2017). Common research questions tested using EMA data often involve whether intraindividual (i.e., within-subject) means or variances in a repeatedly-measured variable such as affect, physical activity, or sleep can predict an individual health-related outcomes such as body mass index or mental health (Brose et al., 2020; Hooker et al., 2020; Jenkins et al., 2018; Von Stumm, 2016). An example research question could explore whether intraindividual variability in affect interacts with the mean level of affect to influence a subject’s physical health outcomes such as cardiovascular function or immune response.

Traditionally, such questions have been addressed by computing intraindividual (i.e., within-subject) means and variances across multiple assessments, typically using standard computational formulas (SCF), such as person-level arithmetic means (iMean) and standard deviations (iSD). These person-level summary statistics are then utilized as regressors in a subsequent regression model to predict or examine their associations with individual health-related outcomes. While these conventional methods are valued for their computational simplicity and ease of interpretation, they also may have limitations and introduce potential biases that could lead to misleading results and compromise the generalizability of the findings.

Prior research has employed the traditional SCF approach to quantify intraindividual variability in sleep by directly calculating aggregated means and standard deviations from daily observations, subsequently using these metrics as predictors to examine associations with physical and mental health outcomes (Slavish et al., 2019). Similarly, other studies have utilized the traditional SCF approach to measure intraindividual variability in physical activity, caloric intake, and sleep as subject-level traits, assessing whether these factors co-vary on a daily basis (Hooker et al., 2020). However, existing literature highlights potential limitations in calculating intraindividual summary statistics using the SCF method. These person-level summary statistics may fail to accurately reflect the true values of means and variability for the variables of interest due to unbalanced data structures, missing data, and the non-continuous nature of data scales (Bauer & Sterba, 2011; Foldnes & Grønneberg, 2022; McNeish, 2021; H. Wu & Leung, 2017). Bland and Altman (1995) criticized using the traditional SCF approach to measure the correlation coefficients in data with a multilevel structure such as repeated observations from several subjects. The center of their argument is that the SCF approach ignores the complexity of the potential relation between between-subject and within-subject variability (Bland & Altman, 1995).

The application of multilevel modeling to intensive longitudinal data provides an alternative approach for assessing intraindividual variability. Recent literature demonstrates that this model-based method for estimating intraindividual, or within-subject, variability has been widely adopted in EMA studies. For example, multilevel models have been used to estimate positive and negative affect across different age groups, revealing that older adults tend to exhibit lower affect variability compared to younger individuals (Röcke et al., 2009; Sliwinski et al., 2009). In addition, building on these developments, the Mixed-Effects Location-Scale (MELS) model has emerged as an extension of the traditional multilevel model, enabling the simultaneous modeling of both mean outcomes and within-subject variances, while accommodating random scale effects across individuals (Hedeker et al., 2008, 2012). The MELS model is increasingly applied to estimate intraindividual means and variability (Dunton et al., 2022; Dzubur et al., 2020; Hedeker & Nordgren, 2013; Hoffman & Walters, 2022; Maher et al., 2024a, 2024b; Nordgren et al., 2020). For instance, Maher et al., (2018a, 2018b) utilized the MELS model to estimate intraindividual means and variability of positive affect, identifying interaction effects between these components on mental health and alcohol consumption among mothers (Maher et al., 2018a, 2018b). Other studies have applied this approach to examine the association between affect variability and physical activity (Do et al., 2024; Maher et al., 2018a, 2018b, 2024a, 2024b) While the MELS model introduces a novel, model-based approach to quantify these key constructs, to the best of our knowledge, no research has yet evaluated whether the MELS approach provides more accurate estimates of intraindividual means and variability compared to the conventional SCF method.

### Standard computational formulas (SCF)

Often, EMA researchers use standard computational formulas (SCF) to generate intraindividual means and variances. These computed values of intraindividual means and variances (i.e., for each person) are then entered as predictors, mediators, or moderators in statistical models. This approach is quite conventional and widely applied in prior studies (Li et al., 2019) because it straightforwardly captures the arithmetic central tendency and the dispersion of the data (Jones et al., 2023).

The mean is calculated by summing the observed numerical values of a variable for a subject and then dividing the total by the number of observations for that subject (Lind et al., 2019). This equation is as below:

(1) $${\text{Mean}}_{i}=\frac{\sum_{j=1}^{n_{i}}\left(y_{ij}\right)}{n_{i}}$$

where $n_{i}$ is the number of observations for the subject and $y_{ij}$ is the measurement y of subject i on occasion j.

Similarly, the standard deviation is another well-known calculating summary that describe the dispersion of the data. The standard deviation (as well as the variance) takes into account how all the values for a subject are distributed. The measure evaluates how the values fluctuate about the mean(Lind et al., 2019). The sample standard deviation is the square root of the sum of squared differences around the mean divided by the sample size minus one.

(2) $${\text{StandardDeviation}}_{i}=\sqrt{\frac{\sum_{j=1}^{n_{i}}{\left(y_{ij}-{\overset{‾}{y}}_{i}\right)}^{2}}{n_{i}-1}}$$

where ${\overset{‾}{y}}_{i}$ is the mean of the measurement y of subject i.

### Potential weakness of using standard computational formulas (SCF) as predictors

Although these summary statistics (i.e., mean and standard deviation or variance) are widely accepted and easily obtained in research (Leertouwer et al., 2022; Moore et al., 2011; Okun et al., 2011; Peacock et al., 2015), several issues that need to be addressed. First, use of the summary statistics in subsequent analyses does not consider that the number of observations for each subject could be different in the EMA data. Ignoring differences in the number of observations treats each summary statistic as if it was equally precise in its estimation across subjects and does not acknowledge uncertainty in the estimates for each subject. When EMA data are missing due to non-response, research design or technical issues, it may introduce biases and potentially lead to invalid conclusions if not appropriately addressed. The traditional SCF approach of calculating the intraindividual summary statistics may ignore potential uncertainty from the unbalanced EMA data, especially from participants who provide fewer data points. Thus, the intraindividual or person-level summary statistics for mean and variances generated through the SCF may not provide equally precise information when the calculated quantities t are entered as predictors of health outcomes in subsequent regression models. In the real-world EMA studies, the numbers of observations per subject can vary quite dramatically (Hedeker et al., 2012; Oleson et al., 2022). As a result, this approach could be problematic and inflate Type I error rates by not recognizing this uncertainty (McNeish, 2021).

Second, this SCF approach assumes that the mean and standard deviation are independent of each other, neglecting the potential correlation between these two parameters. Additionally, the computation of these summary statistics through SCF assumes missing data are MCAR, which often is not the case with real-world EMA data where participants may be more likely to miss EMA prompts during busy time such as when with other people or at work. For example, these types of missing EMA data patterns are frequently characterized by MAR or MNAR (Ziesemer et al., 2020). The failure to meet the MCAR assumption can lead to significant challenges in determining accurate values for the means and variances, leading to biases in the estimation of the associations of these parameters with other individual-level outcomes in a subsequent regression model (Elliott, 2007; Elliott et al., 2012; McNeish, 2021).

Third, summary statistics from SCF may be biased when considering non-continuous scales of outcome variables, particularly those on an ordinal scale. Ordinal data come from categorical response options that can be ordered or ranked based on a specific attribute such as rating a participant monetary positive affect. In EMA surveys, Likert-type scales, typically ranging from 5 to 8 categories, are frequently employed for self-reported rating items (Shi et al., 2023). SCF to calculate intraindividual means and variances often disregard inherent limitations of these scales, such as the bounded nature at both ends of the response spectrum, thereby neglecting potential ceiling or floor effects. When such scales are confined to a limited number of categories, such as a 5-point scale, the resulting distribution may be skewed, and the variability constrained. SCF do not necessarily account for these limitations, potentially leading to biased estimates of means and variances (Oleson et al., 2022; Wu & Leung, 2017).

### Mixed-effects location-scale models (MELS)

Mixed-effects regression models (MRMs) are a useful approach for analyzing EMA data (Hedeker, 2004) that have the potential to address many of the computational and modeling challenges above. MRMs include random effects into regression models to account for the correlation of the repeated observations within clusters (i.e., observations nested within subjects). Hedeker et al. (2008) developed an extension of the MRM, called the mixed-effects location-scale (MELS) model, that simultaneously estimates within- (intraindividual) and between- (interindividual) person means and variances by incorporating a log-linear model for within-subject variance with random effects (Hedeker et al., 2008). This approach relaxes the homogeneity of variance assumption across clusters or groups (i.e., subjects) in the traditional MRMs and allows each subject to have their own degree of within-subject variance (random scale). The implementation of the MELS model enables the use of empirical Bayes estimates for the random location and scale effects as unit-specific mean and variance parameters in the subsequent stage-2 analysis. This approach leverages information from the entire population of observations, thereby enhancing the precision of individual-level parameter estimates through data-driven shrinkage. Specifically, empirical Bayes methods systematically adjust unit-level estimates toward the overall mean, with the degree of shrinkage adaptively determined by the amount of information available for each unit. This not only stabilizes variance estimates for subjects with sparse data, but also markedly reduces mean squared error and enhances estimation accuracy for units characterized by small sample sizes (Efron, 2009; Williams et al., 2021). Further methodological details regarding the implementation of MELS using Empirical Bayes estimation are thoroughly described in Hedeker and Nordgren (Hedeker & Nordgren, 2013).

In order to obtain the mean from the MELS model, we include a random location effect in the mean sub-model. The simplest equation with no covariates is as below:

(3) $$y_{ij}=\beta_{0}+v_{i}+\epsilon_{ij}$$

where $\beta_{0}$ is the grand intercept and $v_{i}$ represents a subject’s random mean (deviation from grand mean, $\beta_{0}$). $\epsilon_{ij}$ is subject i’s error at time j (deviation of a subject’s observations from their mean and the fixed part of the model). In the mean model (Eq. 3), $v_{i}$ is also referred to as a random location effect in the MELS model. The estimated random location effect can then be used as an alternative to the mean calculated from the traditional SCF approach.

The MELS model also allows a within-subject (WS) variance sub-model simultaneously along with the mean sub-model so we can extract the random scale estimate of the model. The simplest equation with no covariates is as below:

(4) $$\sigma_{\epsilon_{ij}}^{2}=\text{exp}\left(\tau_{0}+\omega_{i}\right)$$

Specifically, a log-linear sub-model for the WS variance is used, allowing fixed and random effects to influence the WS variance. The exponential function ensures a positive multiplicative factor for any finite value of coefficients, and so the resulting variance is positive (Hedeker & Nordgren, 2013). In the WS variance sub-model without covariates (Eq. 4), $\sigma_{\epsilon_{ij}}^{2}$ refers to the WS variance, which is subscripted by i and j to indicate that the value can change depending on the fixed and random parts (e.g., if a time-varying regressor was included then it would vary by i and j). $\tau_{0}$ is the grand intercept of WS variance. The magnitude of $\sigma_{\epsilon_{ij}}^{2}$ indicates how data vary within subjects (consistency/erraticism). In particular, the random scale effect, $\omega_{i}$, allows the WS variance to vary across subjects beyond the contribution of grand mean (or any regressors), and this estimated effect represents the within-subject variance for each subject. Moreover, random effects can be used as predictors of subject-level outcomes using plausible values replications in the subsequent (stage-2) models (Mislevy, 1991; M. Wu, 2005). These are obtained by repeatedly sampling from a person’s posterior distribution, corresponding to their random effects, in order to account for the inherent uncertainty in estimation.

Although Eqs. (3) and (4) are presented without covariates, these could be included so that the random effect estimates would be adjusted or conditional on the sub-model covariates. Finally, the MELS model allows the two random effects (e.g., random location and random scale) to be correlated, and adjusts for this correlation by obtaining the standardized random effect estimates. This can be important for rating scale data where there may be ceiling or floor effects of measurement that induce correlations between a subject’s mean and their variability (Dzubur et al., 2020).

**Fig. 1:**
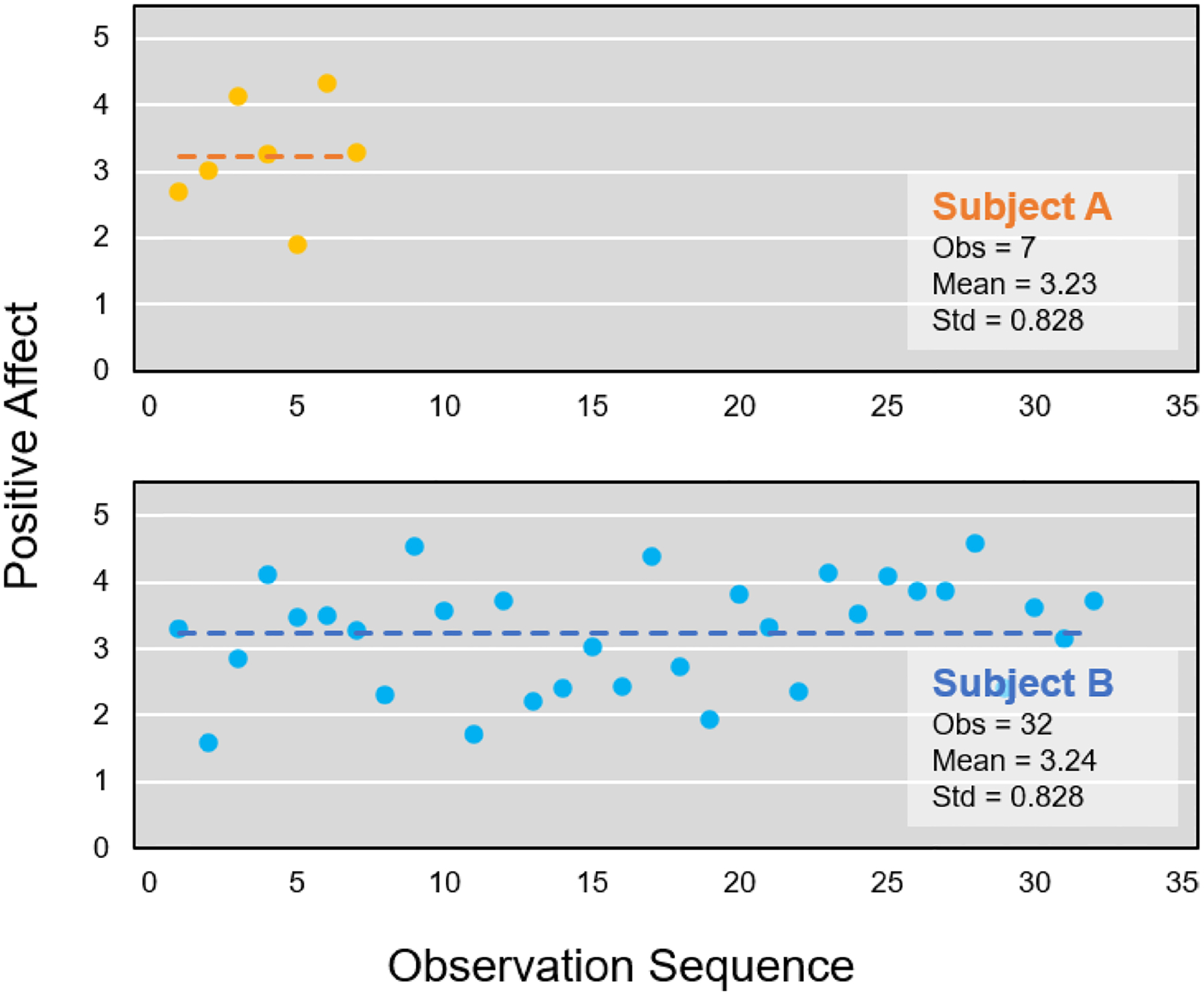
The hypothetical data with two subjects with similar mean and standard deviation but different observations

Figure 1 displays hypothetical data from two subjects to illustrate some of these issues. Notably, both subjects exhibit similar means and variability, yet they differ significantly in their numbers of observations. Specifically, it is evident that Subject B has a greater number of observations than Subject A. These differences would be reflected in the posterior distributions of the random location and scale effects. Namely, Subject A would have a much more dispersed posterior distribution, and his/her plausible value replicates would be more varied than Subject B.

Alternatively, the SCF method treats each summary statistic (i.e., mean and standard deviation) as if it was equally precise in its estimation across these two subjects, even though these two subjects have quite different sample sizes. Also, with few observations (e.g., Subject A in Fig. 1), there is a greater risk that the summary statistics may be biased.

**Fig. 2:**
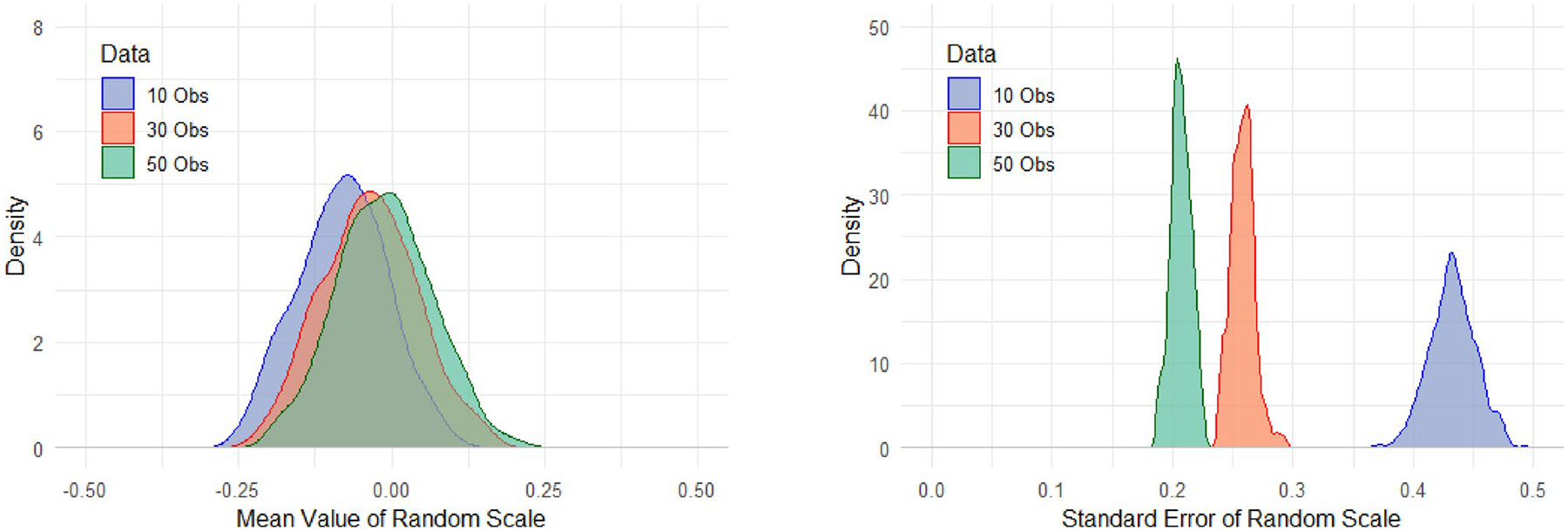
The estimated mean and standard error of subject-level random scale (variance)

In contrast, the MELS model takes into account of the number of observations per subject and estimates the subject-level mean and variability accordingly. This suggests that even when summary statistics appear similar, the MELS model represents the uncertainty in these estimates due to the varying numbers of observations that these summary statistics are based on (Carsey & Harden, 2013). For example, Fig. 2 illustrates a sub-group sample of the subjects with a given standard deviation (e.g., std is around 1) from a simulated dataset. By applying the MELS model, the estimated mean value of random scale is between − 0.25 and 0.25, which indicates that the estimated values of intraindividual variability varies from subject to subject, and it also varies depending on the number of observations.

The random effects estimated by the MELS model are akin to z-scores with mean zero and unit standard deviation. On average, the mean value of random scale in the samples with 30 and 50 observations per subject are more similar to each other, compared to the sample with 10 observations per subject. The standard error of the random scale demonstrates that the MELS model yields a narrower range of estimated random scale values as the number of observations increases. Specifically, samples with 50 observations produce more consistent and accurate estimates of the population parameter (e.g., the true mean of variability) compared to samples with only 10 observations. As previously noted, the SCF method may fail to address biases arising from differences in sample size. Utilizing these biased calculated summary statistics as covariates in predictive models can further distort analyses, potentially resulting in incorrect associations between predictors and outcomes. The primary objective of this study is to use simulated data to compare the predictive coefficients of intraindividual means and variances generated by the SCF and MELS approaches and to evaluate the accuracy of these statistics across the two methods.

## Methods

### Data simulation

To evaluate the use of these parameters derived from traditional SCF approach against those estimated from MELS models, we assessed over 500 simulated datasets, with 500 being the chosen sample size to achieve an estimation error margin of 1% within a 95% confidence interval, our primary metric of interest (Morris et al., 2019).

We utilized a two-step simulation approach for the data. First, we simulated a subject-level outcome to serve as the true outcome $\left(y_{i}\right)$ for the analysis. This subject-level outcome was then modeled using Eq. (5):

(5) $$y_{i}=\beta_{1}{\text{Mean}}_{i}+\beta_{2}STD_{i}+{\text{Error}}_{i}$$

Here, ${\text{Mean}}_{i}$ represents the average for subject $i,STD_{i}$ denotes subject i’s standard deviation, and ${\text{Error}}_{i}$ is the error term for subject i. Here, parameter mean, standard deviation and error were assumed to follow a normal distribution. The coefficients $\beta_{1}$ and $\beta_{2}$, representing the influence of the subject’s mean and standard deviation, were both set to 0.5 to reflect their true impact in the simulated data. This specification ensures that the subject-level outcome $\left(y_{i}\right)$ is a function of the true subject’s mean, standard deviation, and error, with both mean and standard deviation exerting an equal and specified effect size (true value = 0.5) on the outcome.

Second, we generated stage-1 observation-level outcomes $\left(y_{ij}\right)$. We constructed the observation-level outcome $\left(y_{ij}\right)$ to reflect the true subject-level mean $\left(Mean_{i}\right)$, incorporating residuals $\left(R_{ij}\right)$ at the observation level based on subject-level standard deviation variations $\left(STD_{i}\right)$. The general equation is as below:

(6) $$y_{ij}=\sqrt{R_{ij}\times exp\left(STD_{i}\right)}+{\text{Mean}}_{i}$$

This equation is used to simulate the observations within subjects and create the simulated observation-level datasets.

These observation-level datasets are then used in a stage-1 MELS model, from which the random location and scale effects are obtained. Alternatively, the summary statistics from traditional SCF approach are directly calculated from the simulated data. These estimated or calculated values will be inserted back in Eq. 5 to generate the estimated coefficients of mean level $\left(\hat{\beta_{1}}\right)$ and variability $\left(\hat{\beta_{2}}\right)$ so we can compare these estimated coefficients with the true coefficients (0.5) and indirectly assess the performance of mean and variability between SCF and MELS approaches.

### Five data simulation scenarios

To systematically assess how the SCF method and the (stage-1) MELS model approach differ in estimating true means and variability from observation-level data, and in predicting a (stage-2) subject-level outcome variable, we constructed five scenarios:

### Varying numbers of EMA observations across subjects

This scenario examined the impact of unequal numbers of observations per subject (i.e., unbalanced datasets), reflecting realistic EMA data structures. We randomly assigned either 10 or 30 observations to each subject within the datasets. Three conditions were simulated based on the ratio of subjects with 10 to 30 observations (75% vs. 25%, 50% vs. 50%, and 25% vs. 75%). 500 datasets were produced for each condition, each with 300 subjects. An addition of a splitting procedure was set to generate varying observation counts within subjects. The two-step simulation process adhered to Eqs. 5 and 6, with the parameters for subject-level mean, standard deviation, and error terms assumed to follow a normal distribution with a mean of 0.

### Varying mean-to-variance ratios

In real-life EMA data, variables often differ substantially in their underlying mean-to-variance ratios. To reflect this feature, we used simulated data to represent the impact of varying mean-to-variance ratios to reflect the differing relative contributions of mean and variance and evaluate the performance between the SCF and MELS approaches. Datasets were generated with five distinct mean-to-variance (location/scale) ratios: 4.00, 2.00, 1.00, 0.50, and 0.25, such that higher ratios represent a greater contribution of the mean relative to variance. This scenario allowed us to assess whether the SCF and MELS approaches display differential performance when intraindividual variance is relatively small or large compared to the intraindividual mean. To minimize confounding, we fixed the number of subjects (300), the number of observations per subject (30), and the number of simulated datasets (500) across all conditions.

### Varying the proportion of missingness assuming missing completely at random

Recognizing that missing data frequently occur in EMA studies (Courvoisier et al., 2012; Dzubur et al., 2018; Ponnada et al., 2022), this scenario varied the missing data proportion to evaluate the SCF method and MELS model’s robustness when estimating intraindividual means and variances. We implemented three levels of missingness: 10%, 20%, and 30%, all within the Missing Completely at Random (MCAR) framework to the simulation procedure described in Scenario 1. Within MCAR, the probability of data being missing is independent of both the true values of the missing observations and any other measured variables, including covariates. To create a controlled setting, we applied a random seed to simulate a simple MCAR mechanism, ensuring no associations between missingness, the dependent variable, or time-related covariates (Fitzmaurice et al., 2012; Hedeker & Gibbons, 2006; Little, 1995). Under these conditions, the subject-level means and variances remained unbiased. For each missingness level, we simulated data with a constant sample size of 300 subjects. The number of observations per subject varied depending on the specified missing rate, leading to differences in observation counts across subjects. For instance, in the 30% missingness condition, the number of observations per subject ranged from 14 to 29 $\left(M_{\text{obs}}=21.22\right)$. A total of 500 simulated datasets were generated for each missingness condition to ensure robust evaluation of the methods.

### Varying the proportion of missingness assuming missing at random

In this scenario, we examined the effects of varying levels of missingness on the precision of estimated intraindividual means and variability, assuming a Missing at Random (MAR) mechanism. Under MAR, the probability of data being missing depends on observed variables but is independent of the missing data itself. Specifically, we modeled missingness as conditional on subject-level variability (an observed covariate) and a random seed, while ensuring it remained unrelated to unobserved covariates. This condition more accurately reflects the patterns of missing data commonly observed in EMA research (Hedeker & Gibbons, 2006). For instance, subjects may exhibit higher missingness on days when their emotional or health states are lower (e.g., a reduced mean outcome level) or their daily schedules are less stable (e.g., elevated variability in outcomes). Similar to the previous scenario, we fixed the sample size at 300 subjects and adjusted the number of observations to reflect missingness rates of 10%, 20%, and 30%. Each condition was evaluated using 500 simulated datasets, and the outcomes followed the same simulation methodology described in Scenario 1. Please see the appendix for more details.

### Varying the number of categories of ordinal scale responses

In the final scenario, we shifted focus to the outcome variable’s distribution, transitioning from a normally distributed outcome (as in scenarios 1–4, mean equal to 0) to a discrete scale distribution, mirroring common EMA survey formats ordinal scales (e.g., rating happiness from 1 to 5). This shift aims to discern whether the SCF method and MELS model can yield consistent results under varying discrete distribution conditions. Datasets were generated with ordinal responses comprising 5, 6, 7, and 8 categories. The MELS model readily estimated random location and scale effects in all scenarios with continuous outcomes (e.g., Scenario 1 to 4); however, challenges emerged when applying MELS to ordinal data. Specifically, some simulated datasets produced a substantial proportion of subjects exhibiting minimal within-person variation, particularly when the number of response categories was small (e.g., 5-category scales). Limited variability in such cases can hinder the estimation of the random scale components, leading to computational instability and non-convergence of the MELS model. These convergence difficulties were most pronounced in datasets with fewer response categories.

To ensure an adequate number of converged models for evaluating bias and coverage, we increased the number of simulated datasets up to 2,000 per ordinal-scale condition. Across datasets with varying ordinal scale ranges, the proportion of successfully converged MELS model estimations ranged from 26% for outcomes with 5 categories to 82.6% for those with 8 categories. For comparability with earlier scenarios, we held constant the number of subjects (300), the number of observations per subject (30), and the total number of successfully simulated datasets (n = 500) for both the SCF method and the MELS model approaches.

**Fig. 4:**
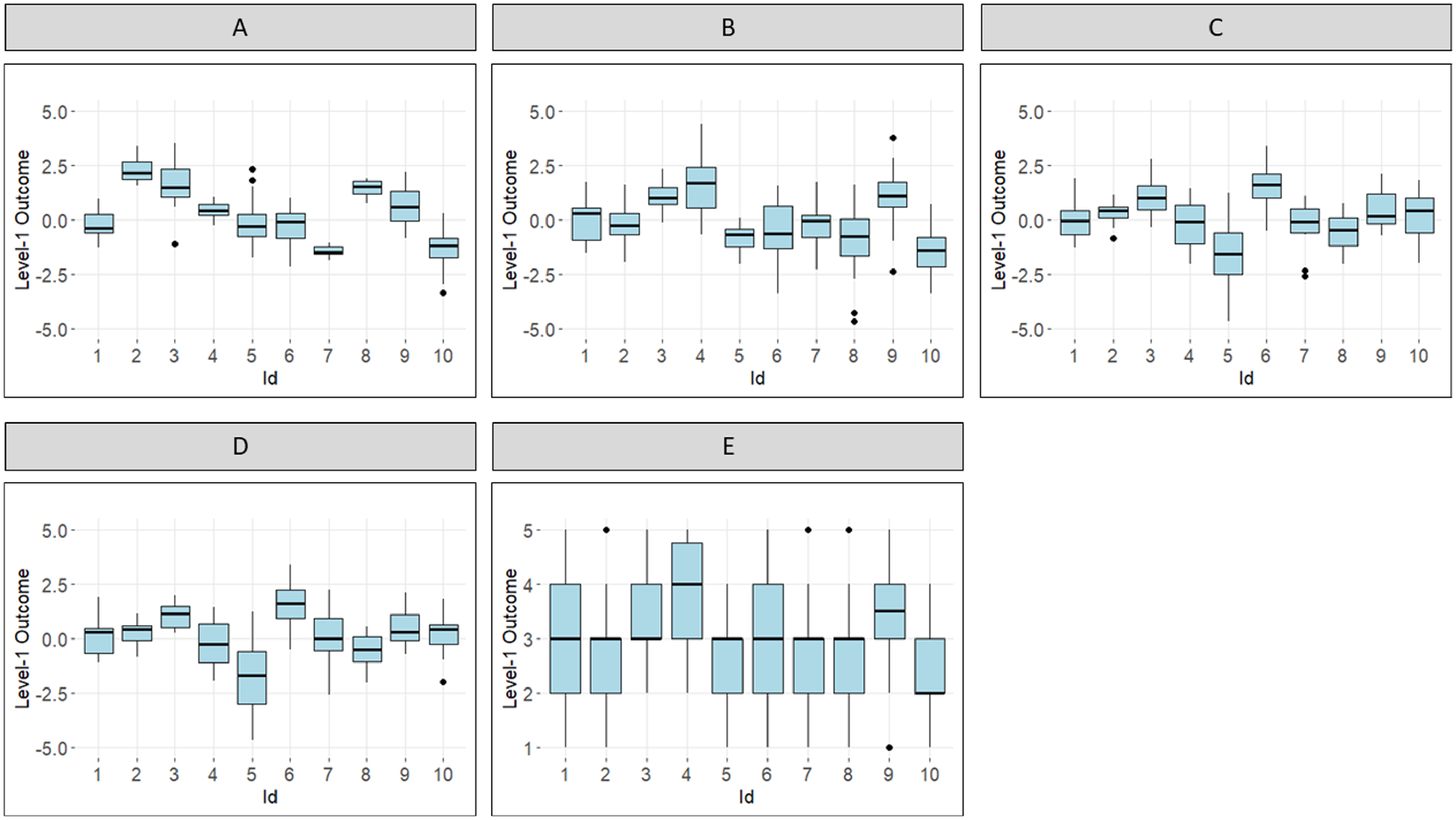
Observation-level (Level-1) outcome in five scenarios. *Note*: The diagrams illustrated the datasets as follows: **A** Varying numbers of EMA observations across subjects; **B** Varying mean-to-variance ratios; **C** Varying the proportion of missingness assuming Missing Completely at Random; **D** Varying the proportion of missingness assuming Missing at Random; (E) Varying the number of categories of ordinal scale responses

The example of these five simulation scenarios is illustrated as Fig. 4.

### Data examination: bias and coverage

To evaluate the SCF method against the MELS model approach, first the SCF method summarized the mean and STD for each subject using Eqs. 1 and 2 across the simulated observation-level datasets. Additionally, we derived estimated subject-level random effects for location (equivalent to mean) and scale (equivalent to variability) using the MELS models within the same dataset in stage-1 models. Consequently, each subject in a single dataset was characterized by four parameters mean, STD, random location, and random scale. We generated over 500 such datasets to ensure the stability and reliability of these parameters.

Then, these subject-level parameters (mean/location and STD/scale) served as predictors in stage-2 linear regression models to predict the true subject-level outcome $\left(y_{i}\right)$. We compared the two approaches by examining the bias and coverage of the predictive coefficients of mean $\left(\hat{\beta_{1}}\right)$ and STD $\left(\hat{\beta_{2}}\right)$ with true coefficients set at 0.5 ($\beta_{1}$ and $\beta_{2}$). Here, “bias” refers to the discrepancy between the estimated parameters and their true values, while “coverage” measures the likelihood that a 95% confidence interval will capture the true parameter value. All simulations and analyses were conducted using SAS 9.4.

## Results

Below are results comparing the SCF versus the MELS model in their ability to approximate the true mean and variability (standard deviation) within simulated datasets across varied scenarios.

### Scenario 1: Varying numbers of EMA observations across subjects

In this scenario, the number of observations per subject was varied, namely either 10 and 30 per subject. For the MELS model, biases for means narrowed from −0.011 in the 75% (10 observations) and 25% (30 observations) configuration to −0.005 in the 25%:75% split. For variability, biases shifted from −0.021 to −0.012 when comparing these same configurations. In terms of coverage in the MELS model, means coverage rates slightly decreased from 95.2% in the 75%:25% split to 94.6% in the 25%:75% split, whereas variability coverage increased from 90.8% to 93.6% between these configurations.

When analyzing datasets with a 75% allocation to 10 observations and a 25% allocation to 30 observations, the SCF method exhibited a coefficient bias of −0.085 for means and −0.060 for variability. Coefficient coverage rates for this dataset configuration were 65.6% for means and 82.2% for variability. Conversely, in datasets with a 25% allocation to 10 observations and a 75% allocation to 30 observations, the bias using the SCF method reduced to −0.053 for means and −0.037 for variability, while coverage rates improved to 84.2% for means and 90.2% for variability (Table 1).

### Scenario 2: Varying mean-to-variance ratios

In this scenario, we simulated datasets with mean-to-variance ratios ranging from 4.00 to 0.25, while maintaining a constant sample size of 30 observations per subject and no missing data. When analyzing datasets with a ratio of 4.00 (i.e., larger mean relative to variance), the MELS model demonstrated coefficient biases of −0.015 for the mean and −0.005 for variability, with coverage rates of 79.6% for the mean and 94.2% for variability. In contrast, for datasets with a ratio of 0.25 (i.e., smaller mean relative to variance), the MELS model yielded a mean bias of −0.005, but the bias for variability became more pronounced at −0.21. Coverage rates for the mean improved to 94.0%, while coverage for variability decreased to 81.2%.

For the SCF method, biases in the mean decreased from −0.106 at a ratio of 4.00 to −0.028 at a ratio of 0.25. For variability, biases shifted from −0.033 to −0.131 across these configurations. Correspondingly, mean coverage rates increased from 61.2% at a ratio of 4.00 to 92.0% at a ratio of 0.25, while variability coverage rates dramatically declined from 91.2% to 46.0% for these two ratios, respectively (Table 2).

### Scenario 3: Varying the proportion of missingness assuming missing completely at random

In this scenario, we adjusted the proportion of missing data to study its impact across three distinct missing rates: 10%, 20%, and 30%, all under the Missing Completely at Random (MCAR) condition. Utilizing the MELS model on the dataset with 10% MCAR missingness yielded biases of −0.006 and −0.007 in mean and variability, respectively. For the 30% MCAR missingness, the biases were −0.007 and −0.011, respectively. Coverage rates for the 10% MCAR condition under the MELS model were 91.4% for the mean and 94.0% for variability. For the 30% MCAR condition, these rates were 92.0% and 93.6%, respectively.

Conversely, Employing the SCF method on the dataset with a 10% MCAR missing rate, we observed coefficient biases of −0.029 and −0.039 for mean and variability, respectively. When the missing rate increased to 30%, these biases shifted to −0.036 for the mean and −0.051 for variability. The coefficient coverage metrics for the 10% MCAR condition stood at 91.4% for the mean and 89.6% for variability, while for the 30% MCAR scenario, they were 90.0% and 85.6%, respectively (Table 3).

### Scenario 4: Varying the proportion of missingness assuming missing at random

Beyond the previous analysis of the MCAR condition, we further investigated the behavior of both the SCF and MELS methodologies under the Missing at Random (MAR) scenario. For this, we maintained the same missing rates, set at 10%, 20%, and 30%, within the MAR context. The MELS model, when applied to the 10% MAR dataset, revealed biases of −0.004 and −0.006 for mean and variability, respectively. For the 30% MAR condition, the biases were slightly altered to 0.005 and −0.009. Coverage under the MELS model for the 10% MAR condition was 91.4% (mean) and 94.0% (variability), and for the 30% MAR scenario, they were 91.8% and 92.4%, respectively.

In comparison, using the SCF method on the dataset with a 10% MAR missing rate, coefficient biases were observed at −0.028 for mean and −0.039 for variability. When the MAR missing rate reached 30%, these biases shifted to −0.034 and −0.066, respectively. Coefficient coverage for the 10% MAR scenario was 91.0% (mean) and 88.6% (variability). For the 30% MAR, coverage metrics were 89.0% and 78.6%, respectively (Table 4).

This study seeks to assess the predictive accuracy of intraindividual means and within-subject variances as determined by SCF compared to those derived from a MELS model. The MELS approach uses empirical Bayes estimation of the model random effects for the subject-level means and variances (Hedeker & Nordgren, 2013). Our analysis commenced with a direct comparison between the SCF and MELS methodologies in generating intraindividual means and variances. We then conducted a series of simulations to evaluate the performance (i.e., the accuracy of coefficients) of summary statistics (SCF) and estimates (MELS) concerning these subject-level parameters across a range of scenarios. These simulations help elucidate the relative efficacy of each approach under varying conditions typical of empirical EMA data.

### Scenario 5: Varying categories of ordinal scale responses

In this scenario, we evaluated both methods using datasets with ordinal outcomes ranging from 5 to 8 categories, maintaining a consistent sample size of subjects and observations (300 subjects with 30 observations each). Implementing the MELS model, the 5-ordinal scale dataset displayed biases of −0.020 for the mean and −0.064 for variability. Meanwhile, the 8-ordinal scale dataset presented biases of −0.005 and −0.008 for mean and variability, respectively. In terms of coverage, the 5-ordinal scale dataset exhibited rates of 91.2% for the mean and 81.1% for variability. Comparatively, the 8-ordinal scale dataset showcased superior coverage of 93.5% for the mean and 94.9% for variability.

**Fig. 5:**
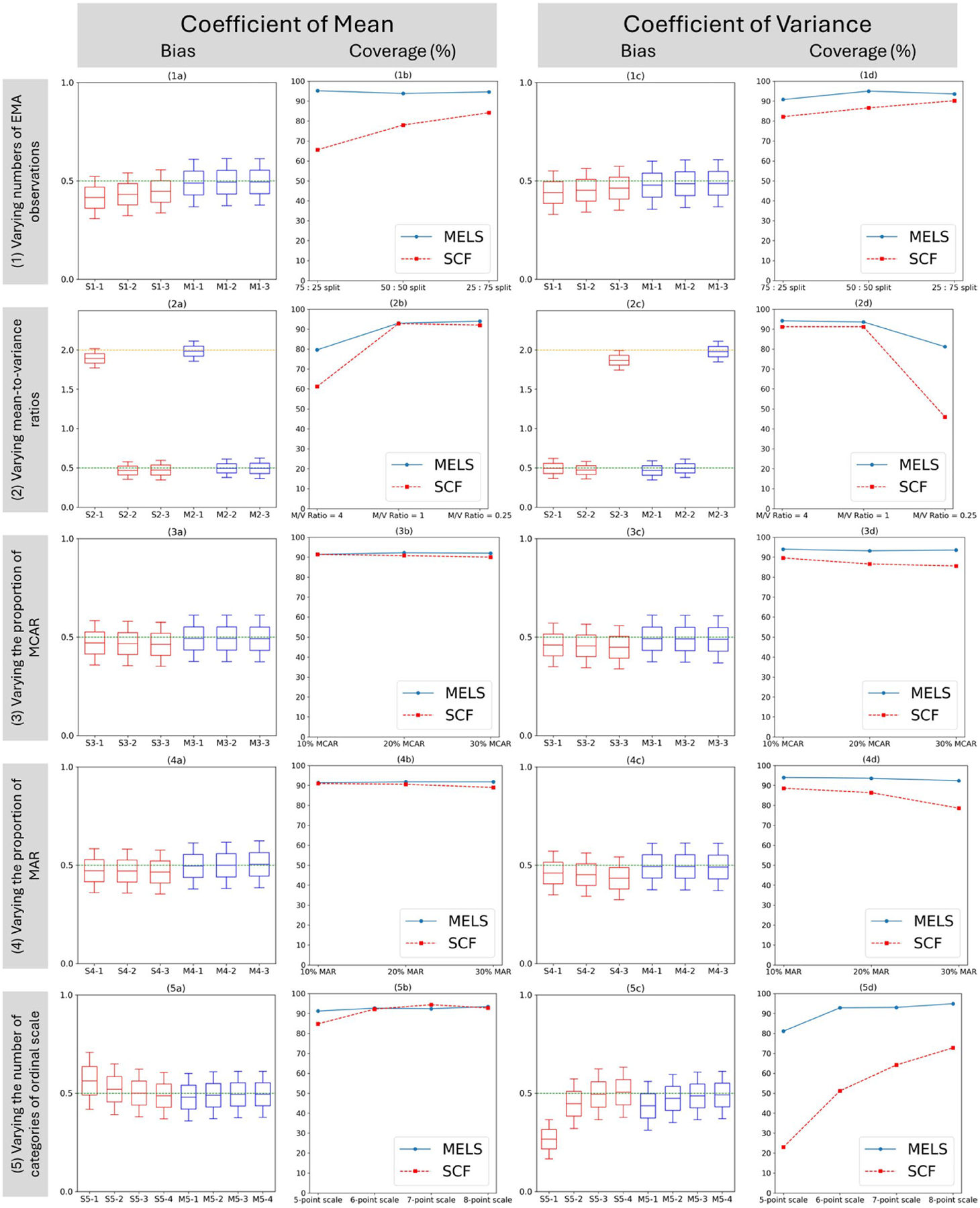
Coefficient bias and coverge of means and variances in all scenarios. Scenario 1 label: S1-1 and M1-1: 75 (10 obs):25 (30 obs) split; S1-2 and M1-2: 50 (10 obs):50 (30 obs) split; S1-3 and M1-3: 25 (10 obs):75 (30 obs) split; Scenario 2 label: S2-1 and M2-1: mean/variance ratio = 4.00; S2-2 and M2-2: mean/variance ratio = 1.00; S2-3 and M2-3: mean/variance ratio = 0.25; Scenario 3 label: S3-1 and M3-1: 10% MCAR; S3-2 and M3-2: 20% MCAR; S3-3 and M3-3: 30% MCAR; Scenario 4 label: S4-1 and M4-1: 10% MAR; S4-2 and M4-2: 20% MAR; S4-3 and M4-3: 30% MAR; Scenario 5 label: S5-1 and M5-1: 5-point scale; S5-2 and M5-2: 6-point scale; S5-3 and M5-3: 7-point scale; S5-4 and M5-4: 8-point scale

When utilizing the SCF method on the 5-ordinal scale dataset, a mean coefficient bias of 0.064 and a variability coefficient bias of −0.234 emerged. Conversely, in the 8-ordinal scale dataset, the mean bias was −0.012 and the variability bias stood at 0.006. coefficient coverage metrics revealed that for the 5-ordinal scale dataset, 84.8% of the true mean and 23.0% of true variability were captured, while for the 8-ordinal scale dataset, these percentages were 92.8% and 72.8%, respectively (Fig. 5, Table 5).

For example, Hooker et al. (2020) used standard deviation to measure intraindividual variability in multiple health behaviors (i.e., physical activity, caloric intake, and sleep duration) over a week, and employed these summary statistics as predictors to examine their association with health outcomes (i.e., body mass index and body fat percentage). This illustrates the utility and widespread adoption of the SCF approach for assessing intraindividual variability in research. However, their study also highlights potential limitations related to subject-level sample size and missing data, which may affect the validity of the results when using the SCF method to calculate means and variability.

### Real-life EMA data example to illustrate the value of comparing subject-level means and variances of positive and negative affect responses in an intensive longitudinal data

To illustrate the differences in calculation and estimation methods, we applied both approaches to a real-life dataset from the Temporal Influences on Movement and Exercise (TIME) study (Wang et al., 2022). The TIME study is a prospective observational investigation that enrolled young adults (ages 18–29) and collected intensive longitudinal data via smartphones and smartwatches over a 12-month period. Participants completed daily ecological momentary assessments (EMAs) using these devices, as previously described. Each day, participants responded to survey prompts assessing affective and other EMA questions before the end of the day. For this analysis, we selected data from the first 50 days following participants’ official enrollment, resulting in a dataset with up to 50 observations per subject to align with the simulation conditions. Due to natural patterns of missing data in real-life study, the resulting dataset mirrors Scenario 1—subjects with varying observations, contributing between 5 and 50 person-days. The final analytic sample comprised 11,008 person-day observations nested within 283 subjects (mean observations per subject = 38.90, SD = 12.67).

Positive affect was measured using three items (e.g., Over the past day, “how happy,” “how energetic,” and “how relaxed” did you feel?), while negative affect comprised six items (“How sad,” “How fatigued,” “How tense,” “How stressed,” “How frustrated,” and “How nervous” did you feel?). All items were rated on a five-point Likert scale, ranging from 1 (not at all) to 5 (extremely). Composite scores for positive and negative affect were calculated by averaging the corresponding items for each subject. Across all subjects and observations, the mean (SD) for positive affect was 2.99 (0.83), and for negative affect, 2.09 (0.82).

Consistent with the simulation study, the subject-level means and variabilities of positive and negative affect were calculated using the SCF method and estimated via the MELS model. To facilitate comparison between the two approaches, we adopted the same stratification strategy as in Scenario 1, categorizing subjects into three groups based on the number of observations: Group 1 (10 or fewer observations), Group 2 (11–30 observations), and Group 3 (31–50 observations). This grouping closely mirrors the conditions of the simulated data, enabling parallel interpretation. Full results are presented in Table 6.

A key distinction between the methods is that the SCF approach provides raw means and standard deviations, whereas the MELS model yields standardized estimates of random location (mean) and random scale (variability). To enable direct comparison, we standardized the SCF-derived means and variabilities, allowing for the evaluation of relative differences across subjects and facilitating a consistent statistical comparison with MELS estimates. Accordingly, the difference between the two methods was calculated as the MELS estimate minus the standardized SCF value.

As shown in Table 6, the differences between SCF and MELS approaches in subject-level means and variabilities of affect were generally small across all groups, especially for the means, whereas more difference was observed for the standard deviations of both positive and negative affect. Post-hoc linear regression analyses were conducted (full results not shown due to space constraints), with group membership coded as dummy variables (Group 1 vs. Group 3, Group 2 vs. Group 3; Group 3 as the reference group) to assess whether observation size influenced the discrepancies between the two approaches. Although most comparisons did not reach conventional statistical significance (p < 0.05), Group 1 (10 or fewer observations) showed a significant negative association with the difference in positive affect variability (β=-0.156, p = 0.023). This finding indicates that, among subjects with limited observations, the SCF method tends to produce higher estimates of positive affect variability than the MELS approach. The correlation coefficients likewise indicated substantially greater disagreement between SCF-calculated and MELS-estimated variability than between their corresponding means, particularly among subjects with fewer than 30 observations.

## Discussion

The current study compared the performance of SCF against the MELS model in terms of their ability to generate values for intraindividual means and variances to be used as predictor of individual level outcomes in subsequent regression models. Our simulations tested five different scenarios varying in the level-1 sample size, mean-to-variance ratios, the patterns of the missingness, and distribution of the outcomes to discern the comparative efficacy of the SCF and MELS methodologies.

When estimating intraindividual means, distinctions between SCF and MELS were relatively minor, with one notable exception: datasets with small numbers of observations per subject. Specifically, when the number of observations per subject fell below 20, MELS emerged as markedly superior, producing robust and precise estimates of subject-level mean (absolute coefficient bias < 0.02; coefficient coverage > 93%), a stark contrast to the SCF method’s outcomes (absolute bias < 0.15; coverage > 50%). Conversely, when evaluating intraindividual variances, the MELS model consistently outperformed the SCF method across the scenarios. Overall, MELS’s random effect estimates of true coefficients of mean and variability tended to be more accurate than those derived from the SCF method.

In EMA research, achieving a balanced design where subjects contribute equal numbers of observations is rare, due to factors such as variability in EMA survey compliance, attrition, and event-contingent designs where surveys are prompted when specific behaviors or contexts are detected. Harrison and his colleagues (2018) suggested that random effect estimation can be unstable if the sample size per subject is highly unbalanced (Harrison et al., 2018). The results underscore that the MELS model, when estimating subject-level means and intraindividual variances, outperforms the SCF approach. The MELS model uniquely partitions variance into components of mean and variability in a model-based approach, and accounts for differences in the number of observations per subject, as well as the correlation of the mean and variance.

Traditional methods, such as the SCF approach, assume uniform precision across all subjects for each summary statistic, such as mean and standard deviation, and that these are independent. In contrast, the MELS model acknowledges the variation in the number of observations per subject. This means that even when summary statistics appear similar, the MELS model reflects the uncertainty in these estimates due to the varying numbers of observations that these summary statistics are based on. When data are nearly balanced, and each subject contributes a large number of observations (exceeding 50), the distinction between SCF and MELS is insignificant. However, for unbalanced datasets, especially when some subjects provide smaller numbers of observations, the MELS model stands out as delivering more precise and dependable outcomes.

Applying both the SCF and MELS model approaches to data with varying mean-to-variance ratios revealed notable differences in the assessment of bias and coverage for the true coefficients of means and variances. When the mean-to-variance ratio was near 1.00—indicating that intraindividual mean and variance contributed equally to the outcome variable—both methods yielded similar accuracy and coverage. However, at the extremes of the ratio spectrum (i.e., very high or very low mean-to-variance ratios), estimation accuracy diverged: the SCF method exhibited substantially greater bias (absolute bias > 0.1) and lower coverage (< 50%) compared to the MELS approach (absolute bias < 0.025; coverage > 80%), particularly in the presence of higher intraindividual variability.

The MELS model offers a model-based estimation framework that accommodates covariates and the association between mean and variance, overcoming limitations inherent in the SCF approach, which cannot adjust for covariates or their associations. Importantly, the MELS model extends traditional mixed-effects models by relaxing the assumption of homogeneity of within-subject variance across occasions and subjects (Blozis et al., 2020; Hedeker et al., 2008). This flexibility allows the MELS model to capture subject-specific variability (random scale), yielding more accurate estimates of variance, especially in datasets characterized by elevated intraindividual variability.

Our findings demonstrate that the MELS model outperforms the SCF method in accurately predicting intra-individual means and variances, particularly under conditions with higher missing data rates (for instance, 30% missingness) in both Missing Completely at Random (MCAR) and Missing at Random (MAR) scenarios. The SCF method operates under the assumption that data are missing completely at random (MCAR)—an assumption that is both stringent and often unrealistic (Little, 2021). Conversely, the MELS model, utilizing full maximum likelihood estimation, allows for missing data to be associated with observed covariates or the observed values of the dependent variable, aligning with the MAR assumption. This more realistic approach makes the MELS model preferable for estimating intraindividual means and variances, particularly in instances where the missing data pattern adheres to the MAR assumption.

Furthermore, treating ordinal scales as continuous variables can lead to bias and poor coverage, suggesting that the optimal range for ordinal categories is up to a 7-point scale for accuracy (Bauer & Sterba, 2011; Wu & Leung, 2017). Our analysis confirms that the granularity of the ordinal scale significantly influences the assessment of intraindividual variance, with this effect being particularly pronounced when using the SCF method. We observed a gradual improvement in the accuracy of predictions for intra-individual mean and variance as the number of ordinal categories increased for both methods. Specifically, in the SCF method, predictions for both intraindividual means and variances were notably less accurate with fewer than six categories on the ordinal scale. The predictive performance became markedly more reasonable as the scale expanded to seven categories. Conversely, the MELS model demonstrated relatively robust estimations for predicting intraindividual means and variances with as few as five ordinal categories. These findings indicate that although the MELS model may encounter computational challenges under conditions of limited ordinal response variability, it nonetheless yields more stable and reliable estimates—particularly in data characterized by a small number of response categories.

In the example of real-life data comparing the SCF method and the MELS model, the differences in estimating intraindividual means and variances varied across subjects depending on the number of available observations. Among subjects with more than 30 observations, both methods yielded highly similar estimates for subject-level means. However, notable discrepancies emerged in the estimation of intraindividual variances, particularly among subjects with 10 or fewer observations. Specifically, the SCF method tended to produce standard deviations for positive affect that were, on average, 40–60% higher in this group, reflecting a pronounced sensitivity to sample size at the observation level as well as to the underlying variability of the measured construct.

These findings, consistent with our simulation results, highlight the limitations of the SCF approach for estimating intraindividual variances—especially when observation counts are low. In contrast, the MELS model employs empirical Bayes estimation for subject-level means and variances, leveraging information from the full dataset to inform individual estimates. Through its shrinkage mechanism, the MELS approach systematically adjusts individual estimates toward the overall mean, with the degree of shrinkage determined by both within-unit sample size and between-unit variability. This process effectively stabilizes variance estimates for subjects with limited data, substantially reduces mean squared error, and improves accuracy for small-sample units (Efron, 2009; Williams et al., 2021). Additionally, the MELS model accommodates random scale effects across subjects, allowing for the recognition of unique within-subject variability and mitigating the risk of variance overestimation (Hedeker et al., 2012).

Ultimately, while the MELS model demonstrates clear advantages in the estimation of intraindividual variances, reliable use of means and variances as regressors in subsequent models depends on robust and unbiased estimation of both parameters in empirical data. Only through such rigorous measurement can subsequent analyses yield valid and meaningful results.

## Conclusion and outlook

Our analyses highlight critical limitations in the use of SCF summary statistics (e.g., means, variance, and standard deviations) within the context of EMA studies where the assumption of equal numbers of observations per subject rarely holds, missing data are common, and ordinal scales with fewer response options are frequently used. The SCF method, while traditionally employed, falls short in several key areas when faced with these inherent challenges of EMA data. Specifically, it inaccurately assumes equal precision across subjects with varying data sizes, does not consider correlated nature of within-subject data, treats summary statistics as fixed rather than estimated quantities with error, and operates under the assumption that missing responses occur completely at random (MCAR). When such summary statistics are used as predictors in stage-2 regression models, this introduces bias in the estimation of regression coefficients, as evidenced by our simulation studies, but also skewed standard errors for the stage 2 regressors, thereby inflating the risk of Type-I errors.

In contrast, the MELS model offers a robust alternative that helps to address many of these issues. This approach can adjust for differences in the number of observations per subject, recognizing unique random scale across subjects and the correlation within subjects’ data, thereby providing more accurate estimates. Moreover, the incorporation of plausible value replications acknowledges and accounts for the inherent uncertainty in estimation, a critical consideration overlooked by the SCF method. Most importantly, by employing full maximum likelihood estimation, the MELS model operates within the MAR assumption for handling missing data (Walters, 2015). This assumption is much more realistic for EMA data than the MCAR assumption, offering a significant improvement over traditional SCF approach.

Overall, current evidence supports the MELS model as a superior analytical strategy for Ecological Momentary Assessment (EMA) data, particularly in studies with unbalanced designs and missing data. The MELS approach yields more reliable and valid inferences by enhancing the precision of estimates and accommodating the complexities inherent in real-world EMA data collection, such as ordinal scale responses and varying mean-to-variance ratios. This represents a significant methodological advancement in behavioral and biostatistics research. The accessibility of the MELS model has also increased substantially, with implementation now available in major statistical packages including SAS (PROC NLMIXED) and R (fastregls) (Gill & Hedeker, 2023; Ma & Hedeker, 2020). Additionally, the MixWILD software provides a free, open-source, and user-friendly desktop GUI specifically designed for the analysis of MELS models in intensive longitudinal data (Dzubur et al., 2020). As the adoption of the MELS approach becomes increasingly feasible, it is crucial to apply this methodology to more nuanced data settings, thereby advancing knowledge in behavioral science.

## Figures and Tables

**Fig. 3:**
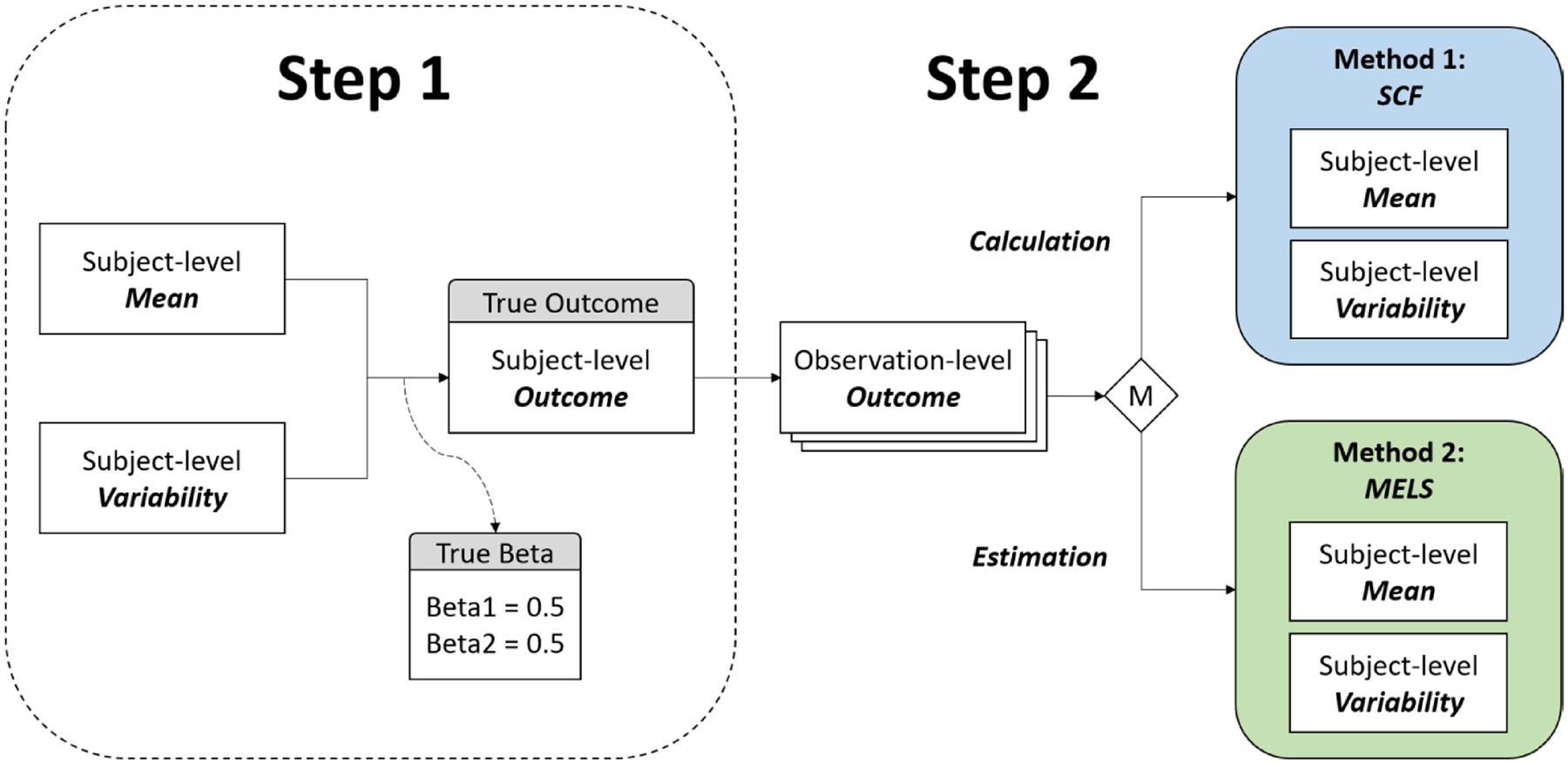
Comparison of model-based and non-model-based approaches for simulating subject-level mean and variability: a two-step simulation framework. *Note*: The figure presents a two-step simulation framework to evaluate the performance of subject-level mean and variability estimation methods. In Step 1, subject-level true outcomes are simulated using subject-level mean, variability, and an error term, with true coefficients (default beta1 and beta2=0.5) assigned to mean and variability to ensure equal influence on the outcome. In Step 2, observation-level outcomes are generated using subject-level parameters. A decision point M determines whether subject-level mean and variability are derived using a non-model-based approach (Method 1: SCF) or a model-based approach (Method 2: MELS). The calculated/estimated parameters from both methods are compared against the true coefficients to assess the accuracy of each approach

**Table 1:** Varying numbers of observations per subject (Change observation numbers per subject unequally in datasets)

Effect and sample size	True value	Est. Mean	Bias	Coverage (%)
*Mixed-effect location-scale model (MELS)*				
Location estimate				
n = 10 or 30 (75: 25 split) for subjects	0.500	0.489	− 0.011	95.20
n = 10 or 30 (50: 50 split) for subjects	0.500	0.494	− 0.006	93.80
n = 10 or 30 (25: 75 split) for subjects	0.500	0.495	− 0.005	94.60
Scale estimate				
n = 10 or 30 (75: 25 split) for subjects	0.500	0.479	− 0.021	90.80
n = 10 or 30 (50: 50 split) for subjects	0.500	0.486	− 0.014	95.00
n = 10 or 30 (25: 75 split) for subjects	0.500	0.488	− 0.012	93.60
*Standard computation formulas (SCF)*				
Mean calculation				
n = 10 or 30 (75: 25 split) for subjects	0.500	0.415	− 0.085	65.60
n = 10 or 30 (50: 50 split) for subjects	0.500	0.432	− 0.068	78.00
n = 10 or 30 (25: 75 split) for subjects	0.500	0.447	− 0.053	84.20
Variance calculation				
n = 10 or 30 (75: 25 split) for subjects	0.500	0.440	− 0.06	82.20
n = 10 or 30 (50: 50 split) for subjects	0.500	0.452	− 0.048	86.60
n = 10 or 30 (25: 75 split) for subjects	0.500	0.463	− 0.037	90.20

**Table 2:** Varying mean-to-variance ratios (Change the ratios of mean-to-variance)

Effect and sample size	True value	Est. Mean	Bias	Coverage (%)
*Mixed-effect location-scale model (MELS)*				
Location estimate				
Mean/Variance coefficient ratio = 4.00	2.000	1.985	− 0.015	79.60
Mean/Variance coefficient ratio = 2.00	1.000	0.991	− 0.009	89.80
Mean/Variance coefficient ratio = 1.00	0.500	0.495	− 0.005	93.00
Mean/Variance coefficient ratio = 0.50	0.500	0.495	− 0.005	93.20
Mean/Variance coefficient ratio = 0.25	0.500	0.495	− 0.005	94.00
Scale estimate				
Mean/Variance coefficient ratio = 4.00	0.500	0.495	− 0.005	94.20
Mean/Variance coefficient ratio = 2.00	0.500	0.495	− 0.005	93.20
Mean/Variance coefficient ratio = 1.00	0.500	0.495	− 0.005	93.60
Mean/Variance coefficient ratio = 0.50	1.000	0.990	− 0.010	91.00
Mean/Variance coefficient ratio = 0.25	2.000	1.979	− 0.021	81.20
Standard computation formulas (SCF)				
Mean calculation				
Mean/Variance coefficient ratio = 4.00	2.000	1.894	− 0.106	61.20
Mean/Variance coefficient ratio = 2.00	1.000	0.946	− 0.054	82.80
Mean/Variance coefficient ratio = 1.00	0.500	0.468	− 0.032	92.80
Mean/Variance coefficient ratio = 0.50	0.500	0.472	− 0.028	91.40
Mean/Variance coefficient ratio = 0.25	0.500	0.472	− 0.028	92.00
Variance calculation				
Mean/Variance coefficient ratio = 4.00	0.500	0.467	− 0.033	91.20
Mean/Variance coefficient ratio = 2.00	0.500	0.468	− 0.032	93.00
Mean/Variance coefficient ratio = 1.00	0.500	0.472	− 0.028	91.20
Mean/Variance coefficient ratio = 0.50	1.000	0.935	− 0.065	79.60
Mean/Variance coefficient ratio = 0.25	2.000	1.869	− 0.131	46.00

**Table 3:** Varying proportions of missing MCAR data (Change the proportions of missingness in the data with assuming missing completely at random

Effect and sample size	True value	Est. Mean	Bias	Coverage (%)
*Mixed-effect location-scale model (MELS)*				
Location estimate				
10% Missing completely at random (MCAR)	0.500	0.494	− 0.006	91.40
20% Missing completely at random (MCAR)	0.500	0.494	− 0.006	92.20
30% Missing completely at random (MCAR)	0.500	0.493	− 0.007	92.00
Scale estimate				
10% Missing completely at random (MCAR)	0.500	0.493	− 0.007	94.00
20% Missing completely at random (MCAR)	0.500	0.491	− 0.009	93.20
30% Missing completely at random (MCAR)	0.500	0.489	− 0.011	93.60
*Standard computation formulas (SCF)*				
Mean calculation				
10% Missing completely at random (MCAR)	0.500	0.471	− 0.029	91.40
20% Missing completely at random (MCAR)	0.500	0.468	− 0.032	90.80
30% Missing completely at random (MCAR)	0.500	0.464	− 0.036	90.00
Variance calculation				
10% Missing completely at random (MCAR)	0.500	0.461	− 0.039	89.60
20% Missing completely at random (MCAR)	0.500	0.456	− 0.044	86.60
30% Missing completely at random (MCAR)	0.500	0.449	− 0.051	85.60

**Table 4:** Varying proportions of missing MAR data (Change the proportion of missingness in the data assuming Missing at Random)

Effect and sample size	True value	Est. Mean	Bias	Coverage (%)
*Mixed-effect location-scale model (MELS)*				
Location estimate				
10% Missing at Random (MAR)	0.500	0.496	− 0.004	91.40
20% Missing at Random (MAR)	0.500	0.500	0.000	91.80
30% Missing at Random (MAR)	0.500	0.505	0.005	91.80
Scale Estimate				
10% Missing at Random (MAR)	0.500	0.494	− 0.006	94.00
20% Missing at Random (MAR)	0.500	0.494	− 0.006	93.60
30% Missing at Random (MAR)	0.500	0.491	− 0.009	92.40
*Standard computation formulas (SCF)*				
Mean calculation				
10% Missing at Random (MAR)	0.500	0.472	− 0.028	91.00
20% Missing at Random (MAR)	0.500	0.470	− 0.030	90.60
30% Missing at Random (MAR)	0.500	0.466	− 0.034	89.00
Variance calculation				
10% Missing at Random (MAR)	0.500	0.461	− 0.039	88.60
20% Missing at Random (MAR)	0.500	0.453	− 0.047	86.40
30% Missing at Random (MAR)	0.500	0.434	− 0.066	78.60

**Table 5:** Varying categories of ordinal scale responses (Change the range of ordinal scale responses)

Effect and sample size	True value	Est. Mean	Bias	Coverage (%)
*Mixed-effect location-scale model (MELS)*				
Mean (Location)				
5-point ordinal scale	0.500	0.480	− 0.020	91.24
6-point ordinal scale	0.500	0.490	− 0.010	92.69
7-point ordinal scale	0.500	0.494	− 0.006	92.41
8-point ordinal scale	0.500	0.495	− 0.005	93.46
Variability (Scale)				
5-point ordinal scale	0.500	0.436	− 0.064	81.14
6-point ordinal scale	0.500	0.474	− 0.026	92.85
7-point ordinal scale	0.500	0.487	− 0.013	93.05
8-point ordinal scale	0.500	0.492	− 0.008	94.92
*Standard computation formulas (SCF)*				
Mean				
5-point ordinal scale	0.500	0.564	0.064	84.80
6-point ordinal scale	0.500	0.521	0.021	92.20
7-point ordinal scale	0.500	0.501	0.001	94.40
8-point ordinal scale	0.500	0.488	− 0.012	92.80
Variability				
5-point ordinal scale	0.500	0.266	− 0.234	23.00
6-point ordinal scale	0.500	0.447	− 0.053	51.20
7-point ordinal scale	0.500	0.495	− 0.005	64.20
8-point ordinal scale	0.500	0.506	0.006	72.80

**Table 6:** Comparison of SCF and MELS approaches in a real-life EMA dataset (Variables of interest—positive and negative affect)

Variable	Group 1: Obs < = 10	Group 2: Obs > 10 and Obs < = 30	Group 3: Obs > 30
	(n = 23)	(n = 26)	(n = 234)
	Average	Average	Average
*MELS (Standardized)*			
Positive affect mean	− 0.320	− 0.562	0.101
Positive affect standard deviation	0.361	0.014	− 0.044
Negative affect mean	− 0.085	0.517	− 0.052
Negative affect standard deviation	0.250	0.145	− 0.036
*Z-score SCF (Standardized)*			
Positive affect mean	− 0.348	− 0.541	0.094
Positive affect standard deviation	0.508	0.024	− 0.053
Negative affect mean	− 0.092	0.503	− 0.047
Negative affect standard deviation	0.275	0.171	− 0.046
*Difference (MELS*—*Z-score SCF)*			
Positive affect mean	0.028	− 0.021	0.006
Positive affect standard deviation	− 0.147	− 0.009	0.009
Negative affect mean	0.007	0.013	− 0.005
Negative affect standard deviation	− 0.025	− 0.027	0.010
*Correlation (MELS vs. SCF)*			
Positive affect mean	0.999	0.970	0.983
Positive affect standard deviation	0.919	0.949	0.963
Negative affect mean	0.987	0.984	0.990
Negative affect standard deviation	0.936	0.829	0.931

## Appendix

### Simulated data—SAS code (missing completely at random (MCAR))

DATA ex_300_30_100pct_MCAR;
* number of datasets;
ndats = 500;
* number of subjects;
ns = 300;
* betas for mean and sd effects;
beta1 =.5;
beta2 =.5;
* random number generator seed;
seed = 12345;
* go over datasets;
subid = 0;
ndat=1;
do while (ndat le ndats);
  * go over subjects;
  n=1;
  do while (n le ns);
   subid = subid+1;
         * get true subject mean and sd - also get error term - all standard normals;
   submean = rannor(seed); subsd = rannor(seed); err = rannor(seed);
         * get stage-2 subject level outcome as fn of true subject mean and sd - and error;
   subz = beta1*submean + beta2*subsd + err;
   * go over repeated obs;
   ni=1;
        nis = 30;
   do while (ni le nis);
                   subnum = ranuni(seed);
           * generate stage-1 observations as random draws from true subject mean and sd;
     y = sqrt(exp(subsd))*rannor(seed) + submean;
           * create a missing completely at random (MCAR) scenario based on random seed;
             if subnum <=.30 then }\textrm{y}=.
      output ex_300_30_100pct;
  ni+1;
   end;
 n+1;
 end;
ndat+1;
end;

The provided SAS code is designed to simulate a multilevel dataset with a Missing Completely at Random (MCAR) mechanism, which is useful for evaluating statistical methods under controlled missing data conditions. Several key elements in the code deserve attention:

- The simulation creates 500 datasets (ndats = 500), each containing 300 subjects (ns = 300). This ensures a sufficiently large sample size to study the statistical properties of the MCAR mechanism and its impact on analysis.
- Each subject has 30 repeated observations (nis = 30). These repeated measures mimic longitudinal or hierarchical data structures commonly seen in research, where observations are nested within individuals.
- For each subject, a true mean (submean) and standard deviation (subsd) are sampled from a standard normal distribution (rannor(seed)), introducing variability across subjects. These values represent underlying individual differences in the simulated data.
- The variable subz represents a stage-2 outcome at the subject level, modeled as a linear combination of the subject’s true mean, standard deviation, and a randomly generated error term (err). The coefficients for this linear combination are specified as beta1 = 0.5 and beta2 = 0.5, highlighting the contribution of the true mean and standard deviation to the outcome.
- For each subject, 30 repeated measurements (y) are generated by drawing from a normal distribution with the true mean (submean) and variance derived from the exponentiated standard deviation (sqrt(exp(subsd))). This structure mimics real-world repeated measures data with subject-specific variability.
- To simulate missing data, approximately 30% of the repeated observations are randomly set to missing (if subnum < = 0.30 then y = .) based on a uniform random number generator (ranuni). This ensures the missingness is unrelated to the observed or unobserved data, fulfilling the MCAR assumption.

The final dataset, ex_300_30_100pct_MCAR, incorporates these hierarchical structures and missing data patterns, making it a robust framework for testing statistical models and methods in the presence of incomplete data. The use of specified random seed values (seed = 12,345) ensures reproducibility of the simulation.

### Simulated data—SAS code (missing at random (MAR))

DATA ex_300_30_100pct_MAR;
* number of datasets;
ndats = 500;
* number of subjects;
ns =300;
* betas for mean and sd effects;
beta1 =.5;
beta2 =.5;
* random number generator seed;
seed = 12345;
* go over datasets;
subid = 0;
ndat=1;
do while (ndat le ndats);
  * go over subjects;
  n=1;
  do while (n le ns);
    subid = subid+1;
         * get true subject mean and sd - also get error term - all standard normals;
    submean = rannor(seed); subsd = rannor(seed); err = rannor(seed);
         * get stage-2 subject level outcome as fn of true subject mean and sd - and error;
    subz = beta1*submean + beta2*subsd + err;
    * go over repeated obs;
    ni=1;
        nis = 30;
    do while (ni le nis);
                    subnum = ranuni(seed);
          * generate stage-1 observations as random draws from true subject mean and sd;
     y = sqrt(exp(subsd))*rannor(seed) + submean;
          * create a missing at random (MAR) scenario based on random seed and observed outcome(sd);
     if subnum <=.6 and subsd >= 0 then y =.;
     output ex_300_30_100pct;
   ni+1;
    end;
 n+1;
 end;
ndat+1;
end;

This SAS code simulates a multilevel dataset incorporating a Missing at Random (MAR) mechanism, a scenario where missingness depends on observed subject-level characteristics. This type of simulation is particularly valuable for assessing statistical methods that account for missing data. The dataset, ex_300_30_100pct_MAR, is created through the following process, with several key elements highlighted for clarity:

- The simulation creates 500 datasets (ndats = 500), each containing 300 subjects (ns = 300). This ensures a sufficiently large sample size to study the statistical properties of the MAR mechanism and its impact on analysis.
- Each subject has 30 repeated observations (nis = 30). These repeated measures mimic longitudinal or hierarchical data structures commonly seen in research, where observations are nested within individuals.
- For each subject, a true mean (submean) and standard deviation (subsd) are sampled from a standard normal distribution (rannor(seed)), introducing variability across subjects. These values represent underlying individual differences in the simulated data.
- The variable subz represents a subject-level outcome, modeled as a linear function of the true mean (submean), true standard deviation (subsd), and an error term (err). The coefficients for this model are specified as beta1 = 0.5 and beta2 = 0.5, quantifying the contributions of the mean and standard deviation to the outcome.
- Each repeated measure (y) is drawn from a normal distribution with a mean equal to the subject’s true mean (submean) and a variance derived from the exponentiated standard deviation (sqrt(exp(subsd))). This step introduces realistic variability within subjects.
- Missingness is introduced under a MAR mechanism, where the probability of a missing observation depends on an observed characteristic, specifically the subject’s standard deviation (subsd).
- A uniform random variable (subnum) is generated for each observation. If subnum < = 0.6 and the subject’s subsd is greater than or equal to 0, the observation is set to missing (y = .). This creates a realistic scenario where missingness depends on an observable trait.

The resulting dataset, ex_300_30_100pct_MAR, is a robust framework for evaluating statistical methods under the MAR assumption. It combines hierarchical data structures (subjects with repeated measures), realistic subject-level variability, and a principled missing data mechanism. By explicitly linking the probability of missingness to observed data (subsd), the code creates a nuanced simulation that mirrors real-world research scenarios, where missingness often depends on measured covariates. This design allows researchers to test and refine analytic techniques for handling missing data in a controlled and reproducible setting.

## References

[R1] Bauer DJ, & Sterba SK (2011). Fitting multilevel models with ordinal outcomes: Performance of alternative specifications and methods of estimation. Psychological Methods, 16(4), 373. 22040372 10.1037/a0025813 PMC3252624

[R2] Bland JM, & Altman DG (1995). Calculating correlation coefficients with repeated observations: Part 2—Correlation between subjects. BMJ, 310(6980), 633. 7703752 10.1136/bmj.310.6980.633 PMC2549010

[R3] Blozis SA, McTernan M, Harring JR, & Zheng Q (2020). Two-part mixed-effects location scale models. Behavior Research Methods, 52(5), 1836–1847. 32043225 10.3758/s13428-020-01359-7

[R4] Brose A, Schmiedek F, Gerstorf D, & Voelkle MC (2020). The measurement of within-person affect variation. Emotion, 20(4), 677. 31008618 10.1037/emo0000583

[R5] Burke LE, Shiffman S, Music E, Styn MA, Kriska A, Smailagic A, Siewiorek D, Ewing LJ, Chasens E, & French B (2017). Ecological momentary assessment in behavioral research: Addressing technological and human participant challenges. Journal of Medical Internet Research, 19(3), Article e77. 10.2196/jmir.7138 PMC5371716 28298264

[R6] Carsey TM, & Harden JJ (2013). Monte Carlo simulation and resampling methods for social science. Sage Publications.

[R7] Courvoisier DS, Eid M, & Lischetzke T (2012). Compliance to a cell phone-based ecological momentary assessment study: The effect of time and personality characteristics. Psychological Assessment, 24(3), 713. 22250597 10.1037/a0026733

[R8] Do B, Hedeker D, Wang W-L, Mason TB, Belcher BR, Miller KA, Rothman AJ, Intille SS, & Dunton GF (2024). Investigating the day-level associations between affective variability and physical activity using ecological momentary assessment. Psychology of Sport and Exercise, 70, Article 102542. 10.1016/j.psychsport.2023.102542 PMC10842154 37805039

[R9] Dunton GF, Wang W-L, Intille SS, Dzubur E, Ponnada A, & Hedeker D (2022). How acute affect dynamics impact longitudinal changes in physical activity among children. Journal of Behavioral Medicine, 45(3), 451–460. 35347520 10.1007/s10865-022-00282-w

[R10] Dzubur E, Huh J, Maher JP, Intille SS, & Dunton GF (2018). Response patterns and intra-dyadic factors related to compliance with ecological momentary assessment among mothers and children. Translational Behavioral Medicine, 8(2), 233–242. 29381785 10.1093/tbm/ibx002 PMC6256954

[R11] Dzubur E, Ponnada A, Nordgren R, Yang C-H, Intille S, Dunton G, & Hedeker D (2020). MixWILD: A program for examining the effects of variance and slope of time-varying variables in intensive longitudinal data. Behavior Research Methods, 52(4), 1403–1427. 10.3758/s13428-019-01322-1 31898295 PMC7406537

[R12] Efron B (2009). Empirical Bayes estimates for large-scale prediction problems. Journal of the American Statistical Association, 104(487), 1015–1028. 20333278 10.1198/jasa.2009.tm08523 PMC2844005

[R13] Elliott MR (2007). Identifying latent clusters of variability in longitudinal data. Biostatistics, 8(4), 756–771. 17267391 10.1093/biostatistics/kxm003

[R14] Elliott MR, Sammel MD, & Faul J (2012). Associations between variability of risk factors and health outcomes in longitudinal studies. Statistics in Medicine, 31(23), 2745–2756. 22815213 10.1002/sim.5370 PMC3470883

[R15] Fitzmaurice GM, Laird NM, & Ware JH (2012). Applied longitudinal analysis. Wiley.

[R16] Foldnes N, & Grønneberg S (2022). The sensitivity of structural equation modeling with ordinal data to underlying non-normality and observed distributional forms. Psychological Methods, 27(4), 541. 33793270 10.1037/met0000385

[R17] Gill N, & Hedeker D (2023). Fast estimation of mixed-effects location-scale regression models. Statistics in Medicine, 42(9), 1430–1444. 36796352 10.1002/sim.9679

[R18] Harrison XA, Donaldson L, Correa-Cano ME, Evans J, Fisher DN, Goodwin CE, Robinson BS, Hodgson DJ, & Inger R (2018). A brief introduction to mixed effects modelling and multi-model inference in ecology. PeerJ, 6, Article e4794. 10.7717/peerj.4794 PMC5970551 29844961

[R19] Hedeker D (2004). An introduction to growth modeling. The Sage handbook of quantitative methodology for the social sciences (pp. 215–234).

[R20] Hedeker D, & Gibbons RD (2006). Longitudinal data analysis. Wiley.

[R21] Hedeker D, Mermelstein RJ, & Demirtas H (2008). An application of a mixed-effects location scale model for analysis of ecological momentary assessment (EMA) data. Biometrics, 64(2), 627–634. 10.1111/j.1541-0420.2007.00924.x 17970819 PMC2424261

[R22] Hedeker D, Mermelstein RJ, & Demirtas H (2012). Modeling between-subject and within-subject variances in ecological momentary assessment data using mixed-effects location scale models. Statistics in Medicine, 31(27), 3328–3336. 22419604 10.1002/sim.5338 PMC3655706

[R23] Hedeker D, & Nordgren R (2013). MIXREGLS: A program for mixed-effects location scale analysis. Journal of Statistical Software, 52(12), 1–38. 23761062 10.18637/jss.v052.i12 PMC3676904

[R24] Hoffman L, & Walters RW (2022). Catching up on multilevel modeling. Annual Review of Psychology, 73(1), 659–689. 10.1146/annurev-psych-020821-103525 34982593

[R25] Hooker SA, Oswald LB, Reid KJ, & Baron KG (2020). Do physical activity, caloric intake, and sleep vary together day to day? Exploration of intraindividual variability in 3 key health behaviors. Journal of Physical Activity and Health, 17(1), 45–51. 31756722 10.1123/jpah.2019-0207 PMC7211555

[R26] Jenkins BN, Hunter JF, Cross MP, Acevedo AM, & Pressman SD (2018). When is affect variability bad for health? The association between affect variability and immune response to the influenza vaccination. Journal of Psychosomatic Research, 104, 41–47. 29275784 10.1016/j.jpsychores.2017.11.002 PMC5777674

[R27] Jones DR, Ruiz JM, Schreier HM, Allison MA, Uchino BN, Russell MA, Taylor DJ, Smith TW, & Smyth JM (2023). Mean affect and affect variability may interact to predict inflammation. Brain, Behavior, and Immunity, 109, 168–174. 36681360 10.1016/j.bbi.2023.01.008 PMC10023429

[R28] Leertouwer Ij., Schuurman NK, & Vermunt JK (2022). Are retrospective assessments means of people’s experiences?: Accounting for interpersonal and intrapersonal variability when comparing retrospective assessment data to ecological momentary assessment data. Journal for Person-Oriented Research, 8(2), 52. 36589927 10.17505/jpor.2022.24855 PMC9773960

[R29] Li H, Chen Z, & Zhu W (2019). Variability: Human nature and its impact on measurement and statistical analysis. Journal of Sport and Health Science, 8(6), 527. 31720063 10.1016/j.jshs.2019.06.002 PMC6835034

[R30] Lind DA, Marchal WG, & Wathen SA (2019). Basic statistics for business & economics. McGraw-Hill.

[R31] Little RJ (1995). Modeling the drop-out mechanism in repeated-measures studies. Journal of the American Statistical Association, 90(431), 1112–1121.

[R32] Little RJ (2021). Missing data assumptions. Annual Review of Statistics and Its Application, 8, 89–107.

[R33] Ma Q, & Hedeker D (2020). Modeling of between-and within-subject variances using mixed effects location scale (MELS) models. 4181. 10.1002/sim.5338 PMC3655706 22419604

[R34] Maher JP, Huh J, Intille S, Hedeker D, & Dunton GF (2018a). Greater variability in daily physical activity is associated with poorer mental health profiles among obese adults. Mental Health and Physical Activity, 14, 74–81.

[R35] Maher JP, Labban JD, Hudgins BL, Hevel DJ, Bittel KM, Kennedy-Malone L, & Hedeker D (2024a). Moving beyond mean levels: Associations between subject-level variability in psychological determinants and physical activity in older adults. Journal of Physical Activity and Health, 22(1), 112–122. 39504953 10.1123/jpah.2024-0350

[R36] Maher JP, Ra CK, Leventhal AM, Hedeker D, Huh J, Chou C-P, & Dunton GF (2018b). Mean level of positive affect moderates associations between volatility in positive affect, mental health, and alcohol consumption among mothers. Journal of Abnormal Psychology, 127(7), 639. 30221951 10.1037/abn0000374 PMC6219753

[R37] Maher JP, Wang W-L, Hedeker D, & Dunton GF (2024b). Temporal stability of behavior, temporal cue-behavior associations, and physical activity habit strength among mothers with school-aged children. Psychology & Health, 39(4), 556–571. 10.1080/08870446.2022.2087875 PMC9792630 35757845

[R38] McNeish D (2021). Specifying location-scale models for heterogeneous variances as multilevel SEMs. Organizational Research Methods, 24(3), 630–653.

[R39] Mislevy RJ (1991). Randomization-based inference about latent variables from complex samples. Psychometrika, 56(2), 177–196.

[R40] Moore M, Kirchner HL, Drotar D, Johnson N, Rosen C, & Redline S (2011). Correlates of adolescent sleep time and variability in sleep time: The role of individual and health related characteristics. Sleep Medicine, 12(3), 239–245. 21316300 10.1016/j.sleep.2010.07.020 PMC3050885

[R41] Morris TP, White IR, & Crowther MJ (2019). Using simulation studies to evaluate statistical methods. Statistics in Medicine, 38(11), 2074–2102. 30652356 10.1002/sim.8086 PMC6492164

[R42] Nordgren R, Hedeker D, Dunton G, & Yang C-H (2020). Extending the mixed-effects model to consider within-subject variance for ecological momentary assessment data. Statistics in Medicine, 39(5), 577–590. 31846119 10.1002/sim.8429

[R43] Okun ML, Reynolds CF III., Buysse DJ, Monk TH, Mazumdar S, Begley A, & Hall M (2011). Sleep variability, health-related practices, and inflammatory markers in a community dwelling sample of older adults. Psychosomatic Medicine, 73(2), 142–150. 21097658 10.1097/PSY.0b013e3182020d08 PMC3106426

[R44] Oleson JJ, Jones MA, Jorgensen EJ, & Wu Y-H (2022). Statistical considerations for analyzing ecological momentary assessment data. Journal of Speech, Language, and Hearing Research, 65(1), 344–360. 10.1044/2021_JSLHR-21-00081 PMC9150728 34910571

[R45] Peacock A, Cash C, Bruno R, & Ferguson SG (2015). Day-by-day variation in affect, arousal and alcohol consumption in young adults. Drug and Alcohol Review, 34(6), 588–594. 25588504 10.1111/dar.12238

[R46] Piot M, Mestdagh M, Riese H, Weermeijer J, Brouwer JM, Kuppens P, Dejonckheere E, & Bos FM (2022). Practitioner and researcher perspectives on the utility of ecological momentary assessment in mental health care: A survey study. Internet Interventions, 30, Article 100575. 10.1016/j.invent.2022.100575 PMC9526140 36193339

[R47] Ponnada A, Li J, Wang S, Wang W-L, Do B, Dunton GF, & Intille SS (2022). Contextual biases in microinteraction ecological momentary assessment (μEMA) non-response. Proceedings of the ACM on Interactive, Mobile, Wearable and Ubiquitous Technologies, 6(1), 1–24. 10.1145/3517259 PMC11759496 39866712

[R48] Röcke C, Li S-C, & Smith J (2009). Intraindividual variability in positive and negative affect over 45 days: Do older adults fluctuate less than young adults? Psychology and Aging, 24(4), 863. 20025402 10.1037/a0016276

[R49] Shi D, Hébert E, & Businelle M (2023). A longitudinal network model to assess affect structures in ecological momentary assessment: Likert and slider response formats may not be equivalent.

[R50] Slavish DC, Taylor DJ, & Lichstein KL (2019). Intraindividual variability in sleep and comorbid medical and mental health conditions. Sleep, 42(6), Article zsz052. 10.1093/sleep/zsz052 PMC6559172 30843059

[R51] Sliwinski MJ, Almeida DM, Smyth J, & Stawski RS (2009). Intraindividual change and variability in daily stress processes: Findings from two measurement-burst diary studies. Psychology and Aging, 24(4), 828. 20025399 10.1037/a0017925 PMC2857711

[R52] Trull TJ, & Ebner-Priemer UW (2009). Using experience sampling methods/ecological momentary assessment (ESM/EMA) in clinical assessment and clinical research: Introduction to the special section. 10.1037/a0017653 PMC4255457 19947780

[R53] Von Stumm S (2016). Is day-to-day variability in cognitive function coupled with day-to-day variability in affect? Intelligence, 55, 1–6.

[R54] Walters RW (2015). Mixed-effects location-scale models for conditionally normally distributed repeated-measures data. The University of Nebraska-Lincoln.

[R55] Wang S, Intille S, Ponnada A, Do B, Rothman A, & Dunton G (2022). Investigating microtemporal processes underlying health behavior adoption and maintenance: Protocol for an intensive longitudinal observational study. JMIR Research Protocols, 11(7), Article e36666. 10.2196/36666 PMC9335174 35834296

[R56] Williams DR, Martin SR, Liu S, & Rast P (2021). Bayesian multivariate mixed-effects location scale modeling of longitudinal relations among affective traits, states, and physical activity. European Journal of Psychological Assessment. 10.1027/1015-5759/a000624 PMC8580300 34764628

[R57] Wu H, & Leung S-O (2017). Can Likert scales be treated as interval scales?—A simulation study. Journal of Social Service Research, 43(4), 527–532.

[R58] Wu M (2005). The role of plausible values in large-scale surveys. Studies in Educational Evaluation, 31(2–3), 114–128.

[R59] Ziesemer K, König LM, Boushey CJ, Villinger K, Wahl DR, Butscher S, Müller J, Reiterer H, Schupp HT, & Renner B (2020). Occurrence of and reasons for “missing events” in mobile dietary assessments: Results from three event-based ecological momentary assessment studies. JMIR mHealth and uHealth, 8(10), Article e15430. 10.2196/15430 PMC7593856 33052123

